# A Treatise on Human Physiology

**Published:** 1875-11

**Authors:** 


*A Treatise on Human Physiology; Designed for the use of Students and Practitioners of Medicine.* By John C. Dalton, M. D. Sixth Edition; Revised and Enlarged. Philadelphia: Henry C. Lea, 1875. Buffalo: T. Butler & Son.

It is something less than four years since we had the pleasure of welcoming the fifth edition of Prof. Dalton's admirable text-book of Physiology, but such has been the demand for the work, and so rapid have been the advances in Physiological Science, that the author has found it necessary to prepare a new edition.

The present edition contains an addition of about fifty per cent. over the last, several new cuts have been introduced, and the whole work has undergone a thorough revision. The new chemical nomenclature and notation have been introduced, and the centigrade system of weights and measures is also employed.

It is in the departments of Physiological Chemistry, and of the Physiology of the Nervous System, that the greatest additions have been made.

In speaking of the functions of the liver, he gives credit to Dr. Murchison, whose views have recently been published in the *Lancet*, and elsewhere. The section relating to the glycogenic function of the liver, has not been modified since the last edition. Of the blood, he says, that human blood cannot with certainty be identified by any means now known to medical science; thus repudiating, and very properly, we think, the very positive testimony of Richardson on this point.

The Physiology of the Nervous System is ably considered, and the illustrations which have been added to this section of the work will be found to materially aid the student in his appreciation of the text.

To attempt an exhaustive review of the sixth edition of Dalton's work, would be at this time a work of supererogation. The profession are sufficiently well acquainted with the work from former editions. We can only say that the work maintains fully its high standard as a text-book, and in saying that we can add no higher praise.

